# ‘Think-aloud’ protocol for ICU rounds: an assessment of information assimilation and rational thinking among trainees

**DOI:** 10.3402/meo.v19.25783

**Published:** 2014-10-16

**Authors:** Shahla Siddiqui

**Affiliations:** Khoo Teck Puat Hospital, Singapore, Email: shahlasi@yahoo.com

For clinical trainees, critical care rounds are often complex and baffling – requiring a sound knowledge of physiology and pharmacology as well as evidence-based medicine. The ICU environment can be especially overwhelming in terms of stress, complexity, and demands. Moreover, applying ‘classroom’ knowledge to a wide spectrum of information obtained from a myriad of sources (lab tests, chart notes, computer displays, and bedside assessments of the patient themselves) can be challenging (1, 2). Presented on rounds, assessment of a critically ill patient is the marriage of knowledge application and rationalization of data to reach a logical explanation and treatment plan. Toward this end, prior work by Pronovost and associated used cognitive task analysis techniques to guide physician-team restructuring and task reallocation in the advent of work hour limitations on house staff (3, 4).

Trainees are required to master these skills early on, and the learning curve can be steep. Too often, a trainee may remain introverted, passive, or ‘lost’ while a situation quickly unfolds. Numerous studies have shown thinking aloud to be superior to directly asking questions when developing a rational thinking strategy. Our aim, then, was to explore the use of a ‘think-aloud’ protocol during ICU rounds, and to assess trainees’ performance and satisfaction following a structured exercise. Our hope was to provide important insights toward rational (rather than random) workflow patterns among trainees in an ICU scenario.

## Methods and participants

ICU rounds, as led by the author in a teaching hospital, served as the study setting. Rounds were conducted without modification, and consent was waived given the routine, anonymous nature of the data. The ICU was a 14-bed surgical unit, and the multidisciplinary team comprised an ICU consultant, medical officers, resident trainees, nurses, pharmacists, and physiotherapists. The study subjects were three trainees from within the anesthesia department (two senior and one junior) and one surgery trainee rotating through the ICU.

Data were collective via direct observations by the PI across two consecutive days. During the assessment of a patient, each trainee was assigned a different task – such as reviewing lab findings, interpreting X-rays, reviewing medications, or performing a clinical exam. At the end of each task, trainees were asked to verbally summarize the results, describing their thought processes and their recognition of abnormal findings. Finally, each trainee was given 10 min to formulate an assessment of the patient. This exercise was repeated for each patient, while clinical care goals and plans were outlined by the consultant/PI.

Twelve observations were made of the four trainees during the 2 days of ICU rounds.

Data collection focused on cognitive processes involved with decision-making, assimilation of facts, situational awareness, attention management, sense-making, problem detection, verbalization, planning, and use of available technology. For each trainee, a self- and peer assessment of the ‘think-aloud’ performance was shared during a debriefing at the end of each day. This is a specific structured protocol where, apart from presenting information in an organized manner, obtained information is assimilated into a rational ‘plan of action’ and verbally presented.

### Concurrent think aloud (CTA) methods

1. Can you assess the (task given)? As you read/observe/examine, please verbalize what you are thinking.
2. Please present your findings in a logical (explanatory) framework.
3. Please point out abnormal findings.
4. Please verbalize which task you are performing as a result of your findings (e.g., ordering a renal panel for low potassium on morning labs).
5. Please summarize the patient's current condition based on your findings.

At the end of each session, the following self-assessment was carried out:

### Debriefing and self-assessment – retrospective think aloud (RTA) method

1. How was your experience?
2. Did you like sharing your thoughts aloud?
3. What were the hindrances?
4. How can this help you prevent errors and better rationalize clinical findings?

## Results

### CTA method

On the first day, the more experienced junior anesthesia doctors were able to give clear, precise, and succinct summaries of the physical and laboratory findings. They spoke confidently – making accurate assessments, using technical/medical terms correctly, and rationally connecting to the patient's ‘story’. Similarly, while some trainees were able to form a logical pattern of their observations, they found it difficult to communicate this. When confronted with abnormal findings or problems, there was hesitation and choppiness in their narratives among even the more expressive trainees. However, this improved on Day 2 after receiving feedback and the self-assessment session.

Despite taking the most time, the least experienced trainee had the most difficulty in expression, the correct use of medical terms, and reading the x-ray. There was poor verbalization, assessment, thought linking, and reaching a conclusion. However, once again, this trainee was able to improve and speak with much more confidence after receiving feedback – even when given a more advanced task on the second day.

### Debriefing and self-assessment – RTA method

The experienced junior anesthesia trainees judged this to be a helpful and meaningful exercise. Although they did not like verbalizing their thought process, they found it easy to do – especially if the clinical findings were normal. However, when faced with a difficult assessment or abnormality, they found it easier to think and then speak – rather than think aloud. Not surprisingly, those more vocal trainees found the experience to be easier and more helpful. One trainee, who assumed everyone was unaccustomed to verbalizing their thoughts, found it particularly useful. Another junior trainee (with a non-medical background) also found the exercise beneficial, but felt shyness and unfamiliarity with the ‘language of medicine’ was a limiting factor.

## Discussion

In this observational study, think-aloud tools were explored to identify cognitive aspects of trainees’ critical care practice and learning. Through verbalizing their thought processes, trainees were able to elucidate those activities engaged in their clinical decision making. Although assessments of cognitive work have been undertaken in ICU teams, as well as among ED members to decrease errors in judgment (5, 6), thus far, the utility of specific ‘think-aloud’ methods have not been explored. Our findings suggest that, when prompted, silent trainees on rounds can verbalize their thoughts into a logical framework and tie it to the patient's narrative; we also found that weaker trainees benefited the most by learning to ‘flow’ their thoughts via speaking. Finally, we found that trainees experienced fewer issues with this exercise when clinical findings were routine; faced with abnormalities, their thinking tended to outpace their speech. When this occurs, cognitive errors and communication breakdowns result as a team encounters a crisis or difficult situation (7, 8).

Several conceptual limitations should be noted. First, think-aloud processes alone may not fully reveal all relevant cognitive processes – some of which may not be retrievable or adequately verbalized. Second, for CTA methods, cognitive processes are quicker than verbal processes, so participants might be thinking about more than they are able to verbally express. Third, CTA is more easily affected by reactivity, and subjects may perform better or worse in completing different tasks. That is, some tasks may seem to be easier to do when CTA is used, since an individual is forced to structure his/her thoughts and actions; in contrast, others – like simultaneously speaking and working on a computer – may appear harder as the cognitive workload increases.

## Conclusion

While doctors generally receive substantial training to communicate with patients and their families, this often neglects communication with peers, colleagues, or other team members. More importantly, think-aloud methods can help to identify strengths and weaknesses in trainees’ clinical decision-making skills. Clearly, far more studies are needed to explore the potentials of this fascinating yet simple tool as it relates to learning in a complex clinical environment and, ultimately, to improved patient care.

*Shahla Siddiqui* Khoo Teck Puat Hospital Singapore Email: shahlasi@yahoo.com
